# Insomnia during the COVID-19 pandemic: Evidence from a Longitudinal Cohort study in Singapore

**DOI:** 10.1192/j.eurpsy.2025.464

**Published:** 2025-08-26

**Authors:** P. Satghare, E. Abdin, S. Shafie, B. Tan, M. Subramaniam

**Affiliations:** 1 Research, Institute of mental health, Singapore, Singapore

## Abstract

**Introduction:**

Since the outbreak of Coronavirus Disease 2019 (COVID-19) pandemic, sleep and mental health of the population have been disrupted worldwide. Predisposing, precipitating, and perpetuating factors of insomnia may signify shared mechanisms that relate insomnia symptoms to psychiatric disorders during stressful periods like pandemic. However, long term repercussions of COVID-19 pandemic on sleep features such as insomnia, and psychological health remains poorly elucidated even after the alleviation of the pandemic measures.

**Objectives:**

The current study aims to identify the longitudinal trajectory of insomnia, in terms of prevalence and associated risk factors, during and post COVID-19 pandemic era among the general population of Singapore.

**Methods:**

Current study consists of longitudinal online and in person surveys involving two-point assessments-Phase1 (during pandemic) from May 2020-June 2021 and Phase2 (post pandemic) from October 2023-August 2024. Singapore residents aged 21 years and above, fluent in English, Chinese or Malay language participated in the study. Participants answered an interviewer-administered questionnaire across both timepoints, including Socio-Demographic information, Insomnia Severity Index, Generalised Anxiety Disorder-7, Physical Health Questionnaire, Depression Anxiety Stress Scales and Covid-19 related stressors-Exposure to Covid-19, current and future perceived risk of infection.

**Results:**

597 participants completed both assessments at phase1 and phase2. The prevalence of insomnia increased from 8.83% in phase2 as compared to 8.24% in phase1 (p<0.00). Those aged 50 years and above (vs 21-34 years) (p<0.02), highest educational attainment of primary school (vs University) (p<0.05), being economically inactive (vs employed) (p< 0.01), having mild levels of anxiety (p<0.007) and having severe levels of depression, anxiety or, stress (p<0.005) was seen to be significantly associated with insomnia.

**Conclusions:**

Insomnia prevalence rose from 8.24% in phase1 to 8.83% in phase2 and was significantly associated with anxiety disorder, psychological distress, and perceived stress among Singapore residents in both phases. These findings could be ascribed to the failure in re-establishment of pre-COVID-19 pandemic norms, social situations and working dynamics that might have led to sleep curtailment and insomnia. Study findings can be utilised to design effective targeted interventions like cognitive behavioural therapy, therapist assisted relaxation and meditation programs to improve sleep and reduce psychological distress. Mentioned interventions can be delivered via smartphone applications enabling easy access, monitoring, delivery, and utilization by the vulnerable groups.

**Disclosure of Interest:**

None Declared

